# Insomnia Severity and Its Demographic and Health-Related Correlates Among Paramedic Students in Saudi Arabia: A Cross-Sectional Study

**DOI:** 10.3390/healthcare14193193

**Published:** 2026-09-28

**Authors:** Ahmed Alobaydullah, Abdullah Alruwaili, Zahra Alabdrabulridha, Moneerah Aljomaan, Sara Aljumah

**Affiliations:** 1 Department of Emergency Medical Services, College of Applied Medical Sciences, King Saud bin Abdulaziz University for Health Sciences, Al Ahsa 31982, Saudi Arabia; 2 King Abdullah International Medical Research Center, Al Ahsa 31982, Saudi Arabia; 3 Department of Emergency Medicine, King Abdulaziz Hospital, Ministry of National Guard Health Affairs, Al Ahsa 31982, Saudi Arabia

**Keywords:** insomnia, sleep quality, insomnia severity index, paramedic students, emergency medical services, medical education, Saudi Arabia

## Abstract

**Background**: Sleep disturbances among healthcare students can negatively affect academic performance and future clinical decision-making. However, limited evidence exists on insomnia among Emergency Medical Services (EMS) students, a group exposed to unique academic and operational stressors. This study assessed insomnia severity and its demographic and health-related correlates among paramedic students in Saudi Arabia. **Methods**: A cross-sectional study was conducted among 238 EMS students in Saudi Arabia. Insomnia severity was assessed using the Insomnia Severity Index (ISI). Descriptive statistics summarized insomnia levels, and bivariate tests and multiple linear regression were used to examine group differences and predictors in SPSS. **Results**: The mean ISI score was 11.8 ± 5.3, indicating subthreshold insomnia among paramedic students. Higher insomnia severity was observed among single students than among married students (12.1 vs. 7.4, *p* = 0.005), among students at private universities (19.3 vs. 11.6, *p* = 0.001), and among those with chronic medical or diagnosed mental illness (*p* < 0.05). No significant differences were found by gender, region, academic level, or dormitory residence (*p* > 0.05). Regression analysis showed that marital status and university type were significant predictors of insomnia symptom severity. **Conclusions**: Insomnia is common among EMS students and may have implications for both student well-being and future clinical performance. Addressing sleep health within EMS education may support better academic outcomes and safer healthcare delivery.

## 1. Introduction

Insomnia is a common sleep disorder characterized by persistent difficulty in initiating or maintaining sleep, or experiencing non-restorative sleep despite adequate opportunity. Insomnia has been classified in the Diagnostic and Statistical Manual of Mental Disorders, Fifth Edition (DSM-5) as a condition that leads to “clinically significant distress or impairment in social, occupational, educational, academic, behavioral, or other important areas of functioning” [1]. It affects approximately 1 in 10 adults and up to 1 in 4 older adults, making it a significant public health concern [2]. Unlike temporary sleep disturbances caused by stress or environmental factors, insomnia is a chronic condition that can negatively impact an individual’s physical, psychological, and social well-being [1,2]. These symptoms are different from the clinical diagnosis. Several behavioral and lifestyle factors contribute to the development of insomnia symptoms. Poor sleep hygiene practices, including excessive caffeine intake, irregular sleep schedules, and prolonged screen exposure, have been strongly associated with increased risk of sleep disturbances [3]. Being a healthcare student is associated with elevated levels of stress, and insomnia symptoms have been closely linked with stress and anxiety in this population [4]. Among paramedics and EMTs, poor sleep has been associated with fatigue and adverse safety outcomes [5]. Therefore, EMS students are at a particularly critical intersection, where they are required to perform academically and prepare for a highly demanding and stressful future career.

Studies have found that healthcare students, including nursing, medical, and allied health students, experience sleep problems at alarmingly high rates. The sleep problems among health care students are not limited to poor sleep quality, but can include insomnia, sleep fragmentation, and decreased overall sleep time. The literature on insomnia among healthcare students paints a similar picture when assessing insomnia among students globally and those in the Middle East region. Chaabane et al. [6] found that insomnia symptoms are prevalent in 46-50% of medical students in Saudi Arabia, with a pooled prevalence range of 30% in Jordan to 59% in Morocco. When investigating the larger undergraduate student population, Spyridonidis et al. [7] found that the estimated global pooled prevalence of insomnia was 46%, with Saudi Arabian students reporting insomnia levels as high as 98.5%. The high levels of insomnia are expected in this population when considering the shift that occurs when entering university life. During this transition, students are first introduced to heightened levels of social pressure, new living arrangements, the academic stress stemming from the unstructured nature of the undergraduate curriculum, and disturbed sleep patterns [8,9].

Emergency Medical Services students are a unique subgroup of university students as they experience high levels of stress and are expected to be in a stressful environment after graduation [10]. To put this issue into perspective, paramedics have a 20% prevalence of Post-traumatic Stress Disorder, a 22% prevalence of generalized anxiety, and a 5.2% suicide risk, which are all significantly higher than other public safety professions such as firefighters and police [11,12,13]. The paramedic student is faced with many unique challenges that are only present at the intersection of being a student at a university and studying a highly stressful profession. Many studies attempted to quantify the level of stress in a similar population such as nursing and medical students; however, to the best of our knowledge, there is a lack of studies that investigate self-reported insomnia symptoms in the EMS students’ population. To address this gap, we conducted this study to assess insomnia symptom severity and its demographic and health-related correlates among paramedic students in Saudi Arabia.

## 2. Materials and Methods

### 2.1. Participants and Procedures

Between January and March 2025, a cross-sectional design was used to measure the severity of insomnia symptoms among Saudi Arabian paramedic students. Participants were recruited from universities that offer paramedic training in Saudi Arabia using convenience sampling. Due to the students’ heavy online presence, an online questionnaire was distributed through social media and university email. Participation was voluntary, and no identifiable information was obtained. Participants were made aware of the study’s aim and provided consent before responding.

### 2.2. Measurements

The questionnaire had two main sections. The initial section collected demographic information such as age and education status. Participants provided basic information including gender, nationality, marital status, region of residence, academic level, university type, GPA, living arrangement, presence of chronic illness, and history of mental illness. This section also included screening questions to ensure that only eligible paramedic students could proceed with the survey (See Supplementary File S1).

The second section applied the Insomnia Severity Index (ISI) to establish sleep issues and insomnia symptom severity. ISI is a brief, reliable, and validated scale commonly used for assessing sleep patterns and disorders, with higher scores indicating more insomnia symptoms. In this study, the original English version of the Insomnia Severity Index [14] was used to evaluate the participants’ sleep quality and insomnia levels. We used the English version of the ISI, as paramedic programs in Saudi Arabia are taught in English. In our sample, the ISI scale showed good internal consistency (Cronbach’s α = 0.81). The ISI includes seven items assessing difficulty falling asleep, difficulty staying asleep, early awakening, satisfaction with sleep, interference with daily functioning, noticeability of sleep problems, and distress related to sleep difficulties. Each item was scored on a five-point Likert scale ranging from 0 (no problem) to 4 (very severe problem). Total ISI scores ranged from 0 to 28 and were categorized as follows: 0–7: No clinically significant insomnia, 8–14: Subthreshold insomnia, 15–21: Moderate clinical insomnia, and 22–28: Severe clinical insomnia. Higher scores indicated greater insomnia severity.

### 2.3. Data Management and Analysis Plan

The data were checked for completeness and analyzed using SPSS (IBM SPSS Statistics, Armonk, NY, USA, Version 26). Continuous variables were reported as mean ± SD, and categorical variables as frequencies and percentages. Normality was assessed, and independent samples *t*-tests or Mann–Whitney U tests were applied accordingly. A multiple linear regression model was developed; the independent variables included in the model were gender, age, nationality, marital status, university type, dormitory residence, chronic medical illness, and being diagnosed with mental illness. *p* < 0.05 was considered statistically significant for all tests. Results were presented in tables and figures.

### 2.4. Ethical Considerations

The research was conducted in accordance with ethical guidelines for human participants and approved by the KAIMRC (MNGHA) IRB, reference number: NRA25/002/5. Informed consent was obtained from all participants, and data were stored securely on a password-protected computer.

## 3. Results

### 3.1. Characteristics of the Study

A total of 238 participants were included in the analysis. Most respondents were male (61.3%) and single (94.5%). The largest student group was in the Eastern region (61.3%), followed by the Western region (24.4%). Regarding academic level, 33.6% were in the fourth year and 26.9% in the third year. Most participants lived with their families during their studies (90.8%), while 10.1% lived in student dormitories. Chronic medical illness was reported by 7.6% of participants, and 6.3% reported being diagnosed with a mental illness (Table 1).

The mean Insomnia Severity Index (ISI) score for the total sample was 11.80 ± 5.33. Mean ISI scores by region ranged from 10.42 ± 6.11 in the Southern region to 13.36 ± 4.94 in the Central region. Across academic levels, mean ISI scores ranged from 11.46 ± 5.55 among fourth-year students to 12.44 ± 4.92 among second-year students. By living arrangement, ISI scores were 11.63 ± 5.39 among those living with family, 13.75 ± 6.16 among those living with colleagues, and 13.43 ± 3.41 among those living alone (Figure 1).

### 3.2. Factors Associated with Insomnia Symptoms Severity

Several variables were significantly associated with insomnia symptoms severity. Single participants reported higher ISI scores than married participants (12.06 ± 5.21 vs. 7.38 ± 5.75; Mann–Whitney U = 786.50, Z = −2.81, *p* = 0.005). Participants attending private universities had higher ISI scores than those attending government universities (19.29 ± 4.79 vs. 11.58 ± 5.19; U = 212.00, Z = −3.33, *p* = 0.001).

Participants with a chronic medical illness reported higher insomnia symptom severity compared with those without chronic illness (15.22 ± 6.09 vs. 11.52 ± 5.18; Mann–Whitney U = 1329.00, Z = −2.32, *p* = 0.020). Similarly, those with a diagnosed mental illness had higher ISI scores than those without (16.00 ± 5.88 vs. 11.49 ± 5.18; U = 964.00, Z = −2.73, *p* = 0.006).

No significant differences were observed by gender (Mann–Whitney U = 7565.50, Z = 1.65, *p* = 0.100). Insomnia symptom severity also did not differ by dormitory residence (11.72 ± 5.29 vs. 12.50 ± 5.76; t = −0.68). One-way ANOVA indicated no significant differences across regions (F(4,233) = 1.03, *p* = 0.392), academic levels (F(4,233) = 0.31, *p* = 0.871), or living arrangements (F(2,235) = 1.31, *p* = 0.272). Pearson correlation analysis showed no significant association between age and insomnia symptoms severity (r = −0.08, *p* = 0.257, *n* = 196).

### 3.3. Regression Analyses

Age was not recorded for 41 participants (17.4%). Participants with and without recorded age did not differ in ISI scores (11.73 ± 5.50 vs. 12.12 ± 4.69; *U* = 3778.00, *Z* = −0.50, *p* = 0.614). Multiple linear regression was conducted on completed cases to identify predictors of insomnia symptom severity. Gender, age, nationality, marital status, university type, dormitory residence, chronic medical illness, and diagnosed mental illness were entered simultaneously (Table 2).

The overall model was significant (F(8,187) = 3.67, *p* = 0.001) and explained 13.6% of the variance in ISI scores (R^2^ = 0.136). Marital status (β = −0.210, t = −2.81, *p* = 0.005) and university type (β = 0.206, t = 2.83, *p* = 0.005) were significant predictors. Other variables were not significant. Diagnostic plots indicated approximate normality of residuals, though variance increased modestly at higher predicted values (Supplementary Figure S1).

## 4. Discussion

The main aim of the study was to assess insomnia symptoms severity among paramedic students in Saudi Arabia and explore associated factors. After assessing the insomnia levels among paramedic students, we found an overall mean ISI score of 11.80, corresponding to a subthreshold level of insomnia [14]. When a group presents with non-clinically significant level of insomnia, an opportunity arises for prevention and education. Paramedics have one of the most stressful jobs in the world, and many studies have found an association between work-related stress among paramedics and severe insomnia [15]. Finding that paramedic students, on average, did not fall within the clinically severe insomnia range may help policymakers and health-profession educators prioritize targeted sleep-health awareness and preventive strategies before symptoms worsen [16]. Several topics for these campaigns can include strategies to manage academic and professional workload, stress management, and healthy lifestyle principles [17].

The population of this study is paramedic students in Saudi Arabia; they do not differ from their peers in other health professions in higher education institutions in Saudi Arabia. In a recent systematic review and meta-analysis, health care students showed a pooled prevalence of 52% [18]. It is important to note that medical students have a significantly higher prevalence of insomnia (35–86%) than student groups similar to paramedics, such as respiratory therapy and Health professions students (9–35%) [18]. Previous studies used various measures of insomnia; regarding studies that used the ISI scale, we find that they had similar proportions of students falling in the subthreshold or moderate ranges (65%) [19]. The presence of these levels of insomnia can be attributed to many underlying issues such as academic pressure, demanding coursework, irregular study schedules, exam-related stress, and technology use at night. However, psychological stress has been reported to be the strongest contributor to experiencing insomnia [20,21,22]. Paramedic students carry the extra burden of emergency care preparation, simulated trauma practical skills training, and clinical exposure to life-threatening injuries and conditions. Many cognitive functions are dependent on good sleep, including attention, good clinical judgment, and appropriate learning capacity [15]. Adequate sleep is particularly important for paramedic students, as future professional practice requires high levels of alertness and rapid decision-making in emergencies.

The association between insomnia levels and demographic factors was mostly consistent with current insomnia literature. For example, our finding that being diagnosed with a chronic illness was associated with higher levels of insomnia is in line with a recent investigation using machine learning analysis methods [23]. Additionally, mental illness is among the most influential factors on insomnia levels among health care students. A study investigated the mental health factors and concluded that it is a clinically important factor for insomnia levels in medical residents. More specifically, insomnia was associated with psychiatric history, depression, and anxiety among the students [24,25]. Regarding marital status, our finding adds to the pool of evidence reporting its significant association with insomnia; although some studies report otherwise, the evidence pointing towards its significance is more common [26,27]. Specifically, the same result was found in a group of respiratory students in Saudi Arabia, where being single was associated with higher levels of insomnia symptoms [28]. The notably higher insomnia scores among students at private universities are a novel observation; however, because this subgroup comprised only seven students, it should be regarded as hypothesis-generating rather than conclusive and needs confirmation in larger samples. We can conclude that even though there are some inconsistencies in the reporting of demographic factors in the insomnia literature, there is a clear pattern of a mix of psychological, health-related, and lifestyle influences [29]. Especially among the subthreshold-to-moderate range insomnia levels, a multifactorial explanation is more suitable rather than insomnia being fully explained by basic demographics alone. This interpretation is consistent with the modest regression model performance in this study, which included mostly demographic factors. Potential contributors to insomnia beyond the demographics include academic stress, workload, sleep hygiene, screen exposure, and lifestyle behaviors.

### 4.1. Implications

Our findings imply that sleep health is an important aspect of student wellbeing and academic performance. Neglecting sleep health among paramedic students may cause loss of concentration, impede their learning, and distort their clinical judgment. To limit the effects of insomnia symptoms and proactively support paramedic students, universities should invest in sleep health education programs, stress management programs, and student wellness initiatives.

### 4.2. Limitations

The cross-sectional nature of the study prevented us from establishing causal inferences, and the convenience sampling and self-reported sleep data may have introduced bias. Also, as an anonymous online survey, the response rate and denominator could not be determined, which may limit representativeness; and the over-representation of Eastern region participants may affect the generalizability of the study. The two significant predictors—marital status and university type—were based on small comparison groups (married, *n* = 13; private university, *n* = 7), so these specific estimates should be interpreted with caution and confirmed in larger samples. Because of a form error, age was not recorded for the first 41 participants. Therefore, the regression model was estimated on complete cases (*n* = 196 of 238).

### 4.3. Future Research Directions

Future research should explore behavioral and lifestyle determinants of insomnia; their absence in our study may partly explain the modest variance explained (13.6%). Additionally, researchers may explore sleep patterns during paramedic training using longitudinal designs and design and test theory-driven interventions to improve sleep quality among paramedic students.

## 5. Conclusions

Paramedic students in our sample reported predominantly subthreshold insomnia, with a subgroup showing more severe symptoms, indicating both a burden worth monitoring and a clear opportunity for early, preventive intervention. Developing targeted health interventions to raise awareness of good sleep practices and support positive attitudes is a priority in this population. Involving students in the design and implementation of these health campaigns is an effective strategy to solidify the importance of this issue. Future studies should investigate the behavioral and lifestyle determinants of insomnia.

## Figures and Tables

**Figure 1:**
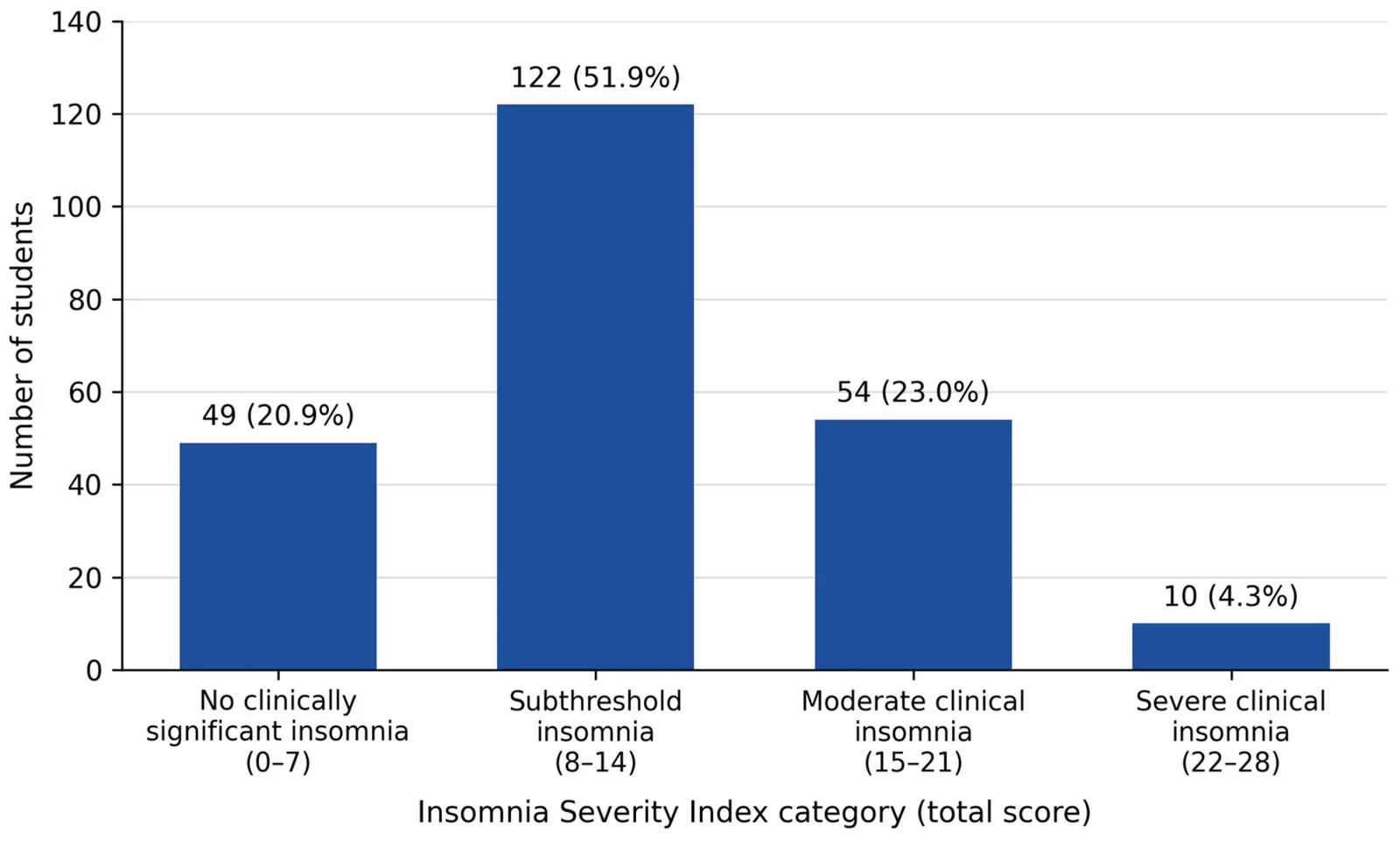
Distribution of insomnia severity categories based on the Insomnia Severity Index (ISI) among paramedic students.

**Table 1:** Participant characteristics and insomnia severity index (ISI) scores.

Variable	Category	*n* (%)	ISI (M ± SD)
Gender	Male	146 (61.3)	11.38 ± 4.93
	Female	92 (38.7)	12.48 ± 5.88
Marital status	Single	225 (94.5)	12.06 ± 5.21
	Married	13 (5.5)	7.38 ± 5.75
Region	Western	58 (24.4)	12.48 ± 5.73
	Eastern	146 (61.3)	11.54 ± 5.09
	Southern	19 (8.0)	10.42 ± 6.11
	Central	14 (5.9)	13.36 ± 4.94
Academic level	1st year	15 (6.3)	11.93 ± 5.69
	2nd year	59 (24.8)	12.44 ± 4.92
	3rd year	64 (26.9)	11.69 ± 5.41
	4th year	80 (33.6)	11.46 ± 5.55
	Internship	20 (8.4)	11.55 ± 5.49
University type	Government	231 (97.1)	11.58 ± 5.19
	Private	7 (2.9)	19.29 ± 4.79
Living arrangement	Family	216 (90.8)	11.63 ± 5.39
	Colleagues	8 (3.4)	13.75 ± 6.16
	Alone	14 (5.9)	13.43 ± 3.41
Dormitory residence	No	214 (89.9)	11.72 ± 5.29
	Yes	24 (10.1)	12.50 ± 5.76
Chronic medical illness	No	220 (92.4)	11.52 ± 5.18
	Yes	18 (7.6)	15.22 ± 6.09
Diagnosed mental illness	No	222 (93.7)	11.49 ± 5.18
	Yes	15 (6.3)	16.00 ± 5.88

ISI, Insomnia Severity Index; M, mean; SD, standard deviation.

**Table 2:** Multiple linear regression analysis predicting insomnia severity index (ISI) scores.

Predictor	B	SE	β	t	*p*	95% CI for B
Constant	6.825	7.442	—	0.917	0.360	−3.69 to 16.87
Gender (Male = 0, Female = 1)	1.168	0.832	0.102	1.404	0.162	−0.46 to 2.83
Age	−0.071	0.236	−0.022	−0.301	0.764	−0.54 to 0.40
Nationality	−0.193	5.224	−0.003	−0.037	0.971	−
Marital status (Single = 0, Married = 1)	−4.994	1.777	−0.210	−2.811	0.005	−8.51 to −1.48
University type	6.055	2.140	0.206	2.830	0.005	1.84 to 10.31
Dormitory residence	−0.348	1.309	−0.019	−0.266	0.791	−3.14 to 2.20
Chronic medical illness	2.119	1.572	0.097	1.348	0.179	−1.16 to 5.34
Diagnosed mental illness	2.087	1.701	0.088	1.227	0.221	−1.23 to 5.51

F(8,187) = 3.67, *p* = 0.001; adjusted R^2^ = 0.136. B, unstandardized coefficient; SE, standard error; β, standardized coefficient; CI, confidence interval.

## Data Availability

The data presented in this study is available on request from the corresponding author. The data is not publicly available due to participant privacy considerations.

## Supplementary Materials

The following supporting information can be downloaded at: https://www.mdpi.com/article/10.3390/healthcare14193193/s1, Figure S1: Model diagnostic; File S1: Syntax.
