# Supplementary material for: Insomnia Severity and Its Demographic and Health-Related Correlates Among Paramedic Students in Saudi Arabia: A Cross-Sectional Study
Source: Healthcare (Basel). 2026 Sep 28;14(19):3193. doi: 10.3390/healthcare14193193 (PMC13665264; doi:10.3390/healthcare14193193)
Supplement: Supplementary file 1 [file healthcare-14-03193-s001.zip › healthcare-4511301-supplementary.pdf]

Insomnia Severity and Its Demographic and Health-Related Correlates Among Paramedic Students in Saudi Arabia: A Cross-Sectional Study

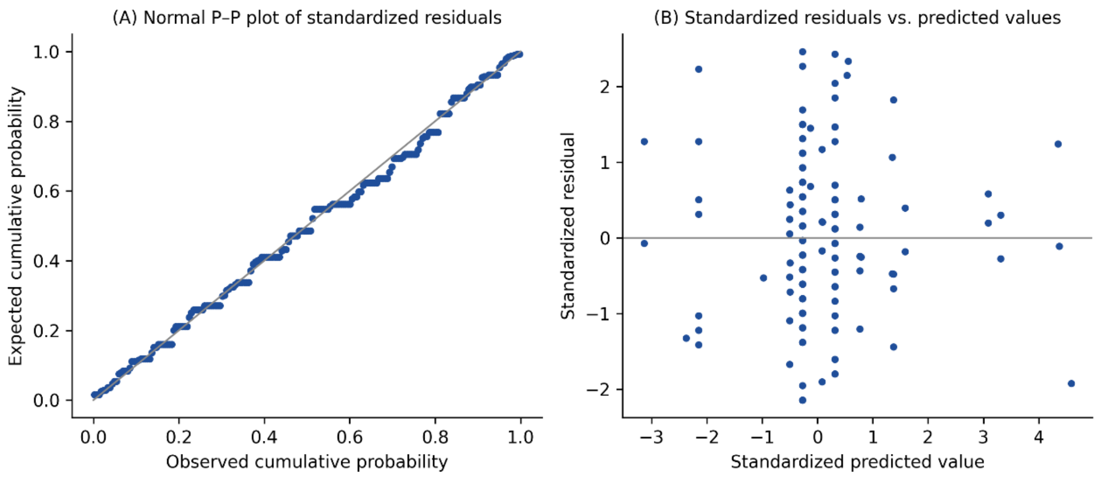

Figure S1. Model diagnostic.

File S1: Syntax

```
.Encoding: UTF-8 *

.(4–0) Create numeric ISI items by extracting the score inside parentheses ---*

.STRING tmp (A200)


= DO REPEAT v

Difficultyfallingasleep

Difficultystayingasleep

Problemwakinguptooearly

HowSATISFIEDdissatisfiedareyouwithyourcurrentsleeppatte

Towhatextentdoyouconsideryoursleepproblemtointerferew

HowNOTICEABLEtoothersdoyouthinkyoursleepingproblemisi

HowWORRIEDdistressedareyouaboutyourcurrentsleepproblem

= nv/

.ISI1_n ISI2_n ISI3_n ISI4_n ISI5_n ISI6_n ISI7_n


.Ensure we are working with a string representation *

.COMPUTE tmp = LTRIM(RTRIM(STRING(v, A200)))


.Extract the digit between '(' and ')', then convert to numeric *

.(4) ... (Works even if the text differs, as long as it contains (0 *

.(COMPUTE nv = NUMBER(SUBSTR(tmp, INDEX(tmp,"(")+1, 1), F1.0


.END REPEAT

.EXECUTE


.Compute ISI total (requires all 7 items) ---*

.COMPUTE ISI_total = SUM.7(ISI1_n, ISI2_n, ISI3_n, ISI4_n, ISI5_n, ISI6_n, ISI7_n)

."(28–0) VARIABLE LABELS ISI_total "Insomnia Severity Index total score

.EXECUTE
```

```
.Quick validation ---*
DESCRIPTIVES VARIABLES=ISI1_n ISI2_n ISI3_n ISI4_n ISI5_n ISI6_n ISI7_n ISI_total
.STATISTICS=MEAN STDDEV MIN MAX/

= DO REPEAT v
Difficultyfallingasleep
Difficultystayingasleep
Problemwakinguptooearly
HowSATISFIEDdissatisfiedareyouwithyourcurrentsleeppatte
Towhatextentdoyouconsideryoursleepproblemtointerferew
HowNOTICEABLEtoothersdoyouthinkyoursleepingproblemisi
HowWORRIEDdistressedareyouaboutyourcurrentsleepproblem
= nv/
.ISI1_n ISI2_n ISI3_n ISI4_n ISI5_n ISI6_n ISI7_n

.(COMPUTE nv = NUMBER(SUBSTR(v, INDEX(v,"(")+1, 1), F1.0

.END REPEAT
.EXECUTE

.COMPUTE ISI_total = SUM.7(ISI1_n, ISI2_n, ISI3_n, ISI4_n, ISI5_n, ISI6_n, ISI7_n)
."(28–0) VARIABLE LABELS ISI_total "Insomnia Severity Index total score
.EXECUTE

DESCRIPTIVES VARIABLES=ISI1_n TO ISI7_n ISI_total
.STATISTICS=MEAN STDDEV MIN MAX/

============================================================*
DEMOGRAPHICS: Convert string categories to numeric safely *
.Creates new variables with _n suffix and keeps originals *
.============================================================*
```

.Gender (1 *

RECODE Gender

(Male"=0")

(Female"=1")

.INTO Gender_n

."VARIABLE LABELS Gender_n "Gender (0=Male, 1=Female)

."VALUE LABELS Gender_n 0 "Male" 1 "Female

.Nationality (2 *

RECODE Nationality

(Saudi"=1")

(Non-Saudi"=2")

.INTO Nationality_n

."VARIABLE LABELS Nationality_n "Nationality

."VALUE LABELS Nationality_n 1 "Saudi" 2 "Non-Saudi

.(Marital status (your variable is named MartialStatus (3 *

RECODE MartialStatus

(Single"=0")

(Married"=1")

.INTO MaritalStatus_n

."VARIABLE LABELS MaritalStatus_n "Marital status (0=Single, 1=Married)

."VALUE LABELS MaritalStatus_n 0 "Single" 1 "Married

.Region (4 *

RECODE Region

(Western"=1")

(Eastern"=2")

(Northern"=3")

(Southern"=4")

(Central"=5")

.INTO Region_n

."VARIABLE LABELS Region_n "Region

VALUE LABELS Region_n

"Western" 1

"Eastern" 2

"Northern" 3

"Southern" 4

."Central" 5

.Academic level (5 *

RECODE AcademicLevel

(st year"=11")

(nd year"=22")

(rd year"=33")

(th year"=44")

(Internship"=5")

.INTO AcademicLevel_n

."VARIABLE LABELS AcademicLevel_n "Academic level

VALUE LABELS AcademicLevel_n

"st year1" 1

"nd year2" 2

"rd year3" 3

"th year4" 4

."Internship" 5

.University type (6 *

RECODE UniversityType

(Government"=1")

(Private"=2")

.INTO UniversityType_n

."VARIABLE LABELS UniversityType_n "University type

."VALUE LABELS UniversityType_n 1 "Government" 2 "Private

.(GPA category (higher = better (7 *

RECODE GPA

(Excellent (above 4.5)"=4")

(Very good (3.75 – 4.5)"=3")

(Good (2.75 – 3.75)"=2")

(Acceptable (2 – 2.75)"=1")

.INTO GPA_n

."VARIABLE LABELS GPA_n "GPA category (higher=better)

VALUE LABELS GPA_n

"Excellent (above 4.5)" 4

"(4.5 – 3.75) Very good" 3

"(3.75 – 2.75) Good" 2

."(2.75 – 2) Acceptable" 1

.Living arrangement (8 *

RECODE Duringyourstudyyoulivedwith

(Family"=1")

(Colleagues"=2")

(Alone"=3")

.INTO LivingWith_n

."VARIABLE LABELS LivingWith_n "Living arrangement during study

VALUE LABELS LivingWith_n

"Family" 1

"Colleagues" 2

."Alone" 3

.Lived in student dorms (9 *

RECODE Duringyourstudydidyouliveinstudentdorms

```
(No"=0")

(Yes"=1")

.INTO Dorms_n

."VARIABLE LABELS Dorms_n "Lived in student dorms (0=No, 1=Yes)

."VALUE LABELS Dorms_n 0 "No" 1 "Yes


.Chronic medical illness (10 *

RECODE Doyouhaveanychronicmedicalillnessese.g.hypertensiond

(No"=0")

(Yes"=1")

.INTO ChronicIllness_n

."VARIABLE LABELS ChronicIllness_n "Chronic medical illness (0=No, 1=Yes)

."VALUE LABELS ChronicIllness_n 0 "No" 1 "Yes


.Diagnosed mental illness (11 *

RECODE Haveyoubeendiagnosedwithamentalillness

(No"=0")

(Yes"=1")

.INTO MentalIllness_n

."VARIABLE LABELS MentalIllness_n "Diagnosed mental illness (0=No, 1=Yes)

."VALUE LABELS MentalIllness_n 0 "No" 1 "Yes


.EXECUTE


============================================================*

Quick check: make sure categories recoded as expected *

.============================================================*

=FREQUENCIES VARIABLES

Gender_n Nationality_n MaritalStatus_n Region_n AcademicLevel_n

UniversityType_n GPA_n LivingWith_n Dorms_n

.ChronicIllness_n MentalIllness_n
```

```
=============================================================*
Recode age categories (string) into numeric midpoint age *
Variable: Whatisyourage *
.=============================================================*

RECODE Whatisyourage
(Under 18"=17")
(21="24 - 18")
(27.5="30 - 25")
(Above 30"=32.5")
.INTO Age_mid

."VARIABLE LABELS Age_mid "Age (midpoint of reported category)
.EXECUTE

.Quick validation *
DESCRIPTIVES VARIABLES=Age_mid
.STATISTICS=MEAN STDDEV MIN MAX/

=============================================================*
Normality testing of ISI_total within comparison groups *
Shapiro–Wilk + Histogram + Normal Q–Q plots *
.=============================================================*

EXAMINE VARIABLES=ISI_total
BY Gender_n Nationality_n MaritalStatus_n Region_n AcademicLevel_n
UniversityType_n GPA_n LivingWith_n Dorms_n
ChronicIllness_n MentalIllness_n
PLOT=HISTOGRAM NPPLOT/
COMPARE GROUPS/
STATISTICS=DESCRIPTIVES/
```

```
CINTERVAL=95/
MISSING=LISTWISE/
.NOTOTAL/

EXAMINE VARIABLES=ISI_total
BY Gender_n
PLOT=HISTOGRAM NPPLOT/
COMPARE GROUPS/
STATISTICS=DESCRIPTIVES/
MISSING=PAIRWISE/
.NOTOTAL/

EXAMINE VARIABLES=ISI_total
BY MentalIllness_n
PLOT=HISTOGRAM NPPLOT/
COMPARE GROUPS/
STATISTICS=DESCRIPTIVES/
MISSING=PAIRWISE/
.NOTOTAL/

EXAMINE VARIABLES=ISI_total
BY ChronicIllness_n
PLOT=HISTOGRAM NPPLOT/
COMPARE GROUPS/
STATISTICS=DESCRIPTIVES/
MISSING=PAIRWISE/
.NOTOTAL/

EXAMINE VARIABLES=ISI_total
BY Region_n
PLOT=HISTOGRAM NPPLOT/
```

```
COMPARE GROUPS/
STATISTICS=DESCRIPTIVES/
MISSING=PAIRWISE/
.NOTOTAL/

EXAMINE VARIABLES=ISI_total
BY MaritalStatus_n
PLOT=HISTOGRAM NPPLOT/
COMPARE GROUPS/
STATISTICS=DESCRIPTIVES/
MISSING=PAIRWISE/
.NOTOTAL/

EXAMINE VARIABLES=ISI_total
BY GPA_n
PLOT=HISTOGRAM NPPLOT/
COMPARE GROUPS/
STATISTICS=DESCRIPTIVES/
MISSING=PAIRWISE/
.NOTOTAL/

EXAMINE VARIABLES=ISI_total
BY AcademicLevel_n
PLOT=HISTOGRAM NPPLOT/
COMPARE GROUPS/
STATISTICS=DESCRIPTIVES/
MISSING=PAIRWISE/
.NOTOTAL/

EXAMINE VARIABLES=ISI_total
BY AcademicLevel_n
```

PLOT=HISTOGRAM NPPLOT/

COMPARE GROUPS/

STATISTICS=DESCRIPTIVES/

MISSING=PAIRWISE/

.NOTOTAL/

.T-tests: ISI_total by binary demographic variables*

(1 0)T-TEST GROUPS=Gender_n

VARIABLES=ISI_total/

.MISSING=ANALYSIS/

(2 1)T-TEST GROUPS=Nationality_n

VARIABLES=ISI_total/

.MISSING=ANALYSIS/

(1 0)T-TEST GROUPS=MaritalStatus_n

VARIABLES=ISI_total/

.MISSING=ANALYSIS/

(2 1)T-TEST GROUPS=UniversityType_n

VARIABLES=ISI_total/

.MISSING=ANALYSIS/

(1 0)T-TEST GROUPS=Dorms_n

VARIABLES=ISI_total/

.MISSING=ANALYSIS/

(1 0)T-TEST GROUPS=ChronicIllness_n

VARIABLES=ISI_total/

.MISSING=ANALYSIS/

(1 0)T-TEST GROUPS=MentalIllness_n

VARIABLES=ISI_total/

.MISSING=ANALYSIS/

.ANOVA: ISI_total by multi-level demographic variables*

ONEWAY ISI_total BY Region_n

STATISTICS DESCRIPTIVES/

.POSTHOC=BONFERRONI/

ONEWAY ISI_total BY AcademicLevel_n

STATISTICS DESCRIPTIVES/

.POSTHOC=BONFERRONI/

ONEWAY ISI_total BY GPA_n

STATISTICS DESCRIPTIVES/

.POSTHOC=BONFERRONI/

ONEWAY ISI_total BY LivingWith_n

STATISTICS DESCRIPTIVES/

.POSTHOC=BONFERRONI/

.Correlation between age and ISI_total*

CORRELATIONS

VARIABLES=Age_mid ISI_total/

PRINT=TWOTAIL/

.MISSING=PAIRWISE/

==*

Non-parametric bivariate tests *

DV: ISI_total *

.==*

------------------------*

(Mann–Whitney U tests (2 groups (1 *

.------------------------*

NPTESTS

INDEPENDENT TEST (ISI_total) GROUP (Gender_n)   MANN_WHITNEY/

MISSING SCOPE=ANALYSIS USERMISSING=EXCLUDE/

.CRITERIA ALPHA=0.05/

NPTESTS

INDEPENDENT TEST (ISI_total) GROUP (Nationality_n) MANN_WHITNEY/

MISSING SCOPE=ANALYSIS USERMISSING=EXCLUDE/

.CRITERIA ALPHA=0.05/

NPTESTS

INDEPENDENT TEST (ISI_total) GROUP (MaritalStatus_n) MANN_WHITNEY/

MISSING SCOPE=ANALYSIS USERMISSING=EXCLUDE/

.CRITERIA ALPHA=0.05/

NPTESTS

INDEPENDENT TEST (ISI_total) GROUP (UniversityType_n) MANN_WHITNEY/

MISSING SCOPE=ANALYSIS USERMISSING=EXCLUDE/

.CRITERIA ALPHA=0.05/

NPTESTS

INDEPENDENT TEST (ISI_total) GROUP (Dorms_n) MANN_WHITNEY/

MISSING SCOPE=ANALYSIS USERMISSING=EXCLUDE/

.CRITERIA ALPHA=0.05/

```
NPTESTS

INDEPENDENT TEST (ISI_total) GROUP (ChronicIllness_n) MANN_WHITNEY/

MISSING SCOPE=ANALYSIS USERMISSING=EXCLUDE/

.CRITERIA ALPHA=0.05/

NPTESTS

INDEPENDENT TEST (ISI_total) GROUP (MentalIllness_n) MANN_WHITNEY/

MISSING SCOPE=ANALYSIS USERMISSING=EXCLUDE/

.CRITERIA ALPHA=0.05/

--------------------------*

(Kruskal–Wallis tests (3+ groups (2 *

.--------------------------*

NPTESTS

K-W TEST (ISI_total) GROUP (Region_n)/

MISSING SCOPE=ANALYSIS USERMISSING=EXCLUDE/

.CRITERIA ALPHA=0.05/

NPTESTS

K-W TEST (ISI_total) GROUP (AcademicLevel_n)/

MISSING SCOPE=ANALYSIS USERMISSING=EXCLUDE/

.CRITERIA ALPHA=0.05/

NPTESTS

K-W TEST (ISI_total) GROUP (GPA_n)/

MISSING SCOPE=ANALYSIS USERMISSING=EXCLUDE/

.CRITERIA ALPHA=0.05/

NPTESTS

K-W TEST (ISI_total) GROUP (LivingWith_n)/
```

```
MISSING SCOPE=ANALYSIS USERMISSING=EXCLUDE/
.CRITERIA ALPHA=0.05/

-------------------------*
(Spearman correlation (Age_mid with ISI_total (3 *
.-------------------------*

NONPAR CORR
VARIABLES=Age_mid ISI_total/
PRINT=SPEARMAN TWOTAIL/
.MISSING=PAIRWISE/

NPAR TESTS
(1 0)MANN-WHITNEY = ISI_total BY Gender_n/
(2 1)MANN-WHITNEY = ISI_total BY Nationality_n/
(1 0)MANN-WHITNEY = ISI_total BY MaritalStatus_n/
(2 1)MANN-WHITNEY = ISI_total BY UniversityType_n/
(1 0)MANN-WHITNEY = ISI_total BY Dorms_n/
(1 0)MANN-WHITNEY = ISI_total BY ChronicIllness_n/
.(1 0)MANN-WHITNEY = ISI_total BY MentalIllness_n/

REGRESSION
DEPENDENT ISI_total/
METHOD=ENTER/
Gender_n
Age_mid
Nationality_n
MaritalStatus_n
UniversityType_n
GPA_n
```

```
Dorms_n
ChronicIllness_n
MentalIllness_n
(95)STATISTICS COEFF OUTS R ANOVA CI/
(10.)CRITERIA=PIN(.05) POUT/
.NOORIGIN/

REGRESSION
(95)STATISTICS COEFF OUTS R ANOVA CI/
(10.)CRITERIA=PIN(.05) POUT/
NOORIGIN/
DEPENDENT ISI_total/
METHOD=ENTER/
Gender_n
Age_mid
MaritalStatus_n
GPA_n
Dorms_n
ChronicIllness_n
.MentalIllness_n

=DESCRIPTIVES VARIABLES
ISI_total
Gender_n Age_mid Nationality_n MaritalStatus_n UniversityType_n GPA_n
Dorms_n ChronicIllness_n MentalIllness_n
.STATISTICS=N MEAN STDDEV MIN MAX/

REGRESSION
(95)STATISTICS COEFF OUTS R ANOVA CI/
(10.)CRITERIA=PIN(.05) POUT/
NOORIGIN/
```

```
DEPENDENT ISI_total/

METHOD=ENTER/

Gender_n

Age_mid

Nationality_n

MaritalStatus_n

UniversityType_n

Dorms_n

ChronicIllness_n

.MentalIllness_n


REGRESSION

(95)STATISTICS COEFF OUTS R CHANGE ANOVA CI/

(10.)CRITERIA=PIN(.05) POUT/

NOORIGIN/

DEPENDENT ISI_total/


METHOD=ENTER/

Gender_n

Age_mid

Nationality_n

MaritalStatus_n

UniversityType_n


METHOD=ENTER/

Dorms_n


METHOD=ENTER/

ChronicIllness_n

.MentalIllness_n
```

```
REGRESSION

(95)STATISTICS COEFF OUTS R CHANGE ANOVA CI/

(10.)CRITERIA=PIN(.05) POUT/

NOORIGIN/

DEPENDENT ISI_total/

METHOD=ENTER Gender_n Age_mid Nationality_n MaritalStatus_n UniversityType_n/

METHOD=ENTER Dorms_n/

.METHOD=ENTER ChronicIllness_n MentalIllness_n/


REGRESSION

DEPENDENT ISI_total/

METHOD=ENTER Gender_n Age_mid Nationality_n MaritalStatus_n UniversityType_n GPA_n Dorms_n /
ChronicIllness_n MentalIllness_n

STATISTICS COLLIN TOL/

PLOT=SCATTER(*ZRESID , *ZPRED)/

.SAVE ZRESID/


REGRESSION

(95)STATISTICS COEFF R ANOVA COLLIN TOL CI/

DEPENDENT ISI_total/

METHOD=ENTER Gender_n Age_mid Nationality_n MaritalStatus_n UniversityType_n GPA_n Dorms_n /
ChronicIllness_n MentalIllness_n

SCATTERPLOT=(*ZPRED,*ZRESID)/

RESIDUALS HISTOGRAM(ZRESID) NPPLOT(ZRESID)/

.SAVE ZPRED ZRESID/
```
